# The impact of Hurricane Katrina, a major natural disaster, on assisted reproductive outcomes through an analysis of 451,848 ART cycles

**DOI:** 10.1038/s41598-021-97402-4

**Published:** 2021-09-09

**Authors:** Olcha Meir, Kuokkanen Satu, Xianhong Xie, Negassa Abdissa, Pal Lubna, Jindal Sangita

**Affiliations:** 1 Albert Einstein College of Medicine, 1300 Morris Park Ave, Block Bldg 627, Bronx, NY 10461 USA; 2 Yale School of Medicine, New Haven, CT USA

**Keywords:** Reproductive biology, Reproductive disorders

## Abstract

To evaluate whether pregnancies conceived via assisted reproductive technology and exposed to Hurricane Katrina (HK), one of the most destructive natural disasters in United States history, were more prone to adverse perinatal outcomes, miscarriages, or sex disparities compared with pregnancies established but not directly exposed to the natural disaster. Retrospective cohort study. Women 18 years and older undergoing fresh ART cycles that resulted in singleton pregnancies and entered in the SART CORS database from 2004 to 2008. Incidence of full-term and preterm delivery, miscarriage rate, infant weight at birth, and infant sex ratio. Total follicle stimulating hormone (FSH) stimulation dosage and number of oocytes retrieved. Between January 2004 and December 2008, a total of 451,848 fresh autologous IVF cycles were recorded in SART CORS, leading to 190,624 pregnancies and 129,499 live births. After controlling for potential confounders, our results demonstrate no association between HK exposure and overall preterm deliveries (< 37 weeks) in women with singleton pregnancies conceived after ART. Other perinatal outcomes such as rate of spontaneous abortion or infant weight at birth in the exposed and unexposed groups were also not associated with HK. A comparison of pre and post disaster sex ratios revealed fewer males were born after HK (51.0% vs. 49.4%), showing a trend of decreased male infant births that was least in part associated with HK (CI 0.81–1.01; p = 0.07). Exposure to HK did not appear to affect perinatal outcomes such as the rate of preterm delivery or the rate of spontaneous abortion. Extreme stress may be a factor that contributes to a reduced male to female secondary sex ratio.

## Introduction

Natural disasters such as hurricanes and earthquakes have been increasing in incidence over the past decade, with 90% of these being droughts, storms, and floods. These events have been expected to increase in frequency as global climates change and as a result, the number of people exposed to such disasters will increase over time^1^. These disasters can cause extreme heightened levels of stress emotionally and/or physically^2–4^. Exposure often leads to a variety of mental health consequences, particularly anxiety-mood disorders and posttraumatic stress disorders^5–8^. In addition, other health implications of natural disasters outside of psychological sequelae have also been an area of investigation. Data from the aftermath of Hurricane Katrina (HK), one of the most destructive natural disasters in the United States history, has been utilized to investigate some of these outcomes.

The hurricane which made landfall on August 25th 2005, primarily affected 5 US Federal Emergency Management Agency (FEMA)-designated states: Texas, Louisiana, Mississippi, Alabama, and Florida, and caused destruction along the gulf coast accounting for an estimated 1,570 deaths of Louisiana residents alone and over $40 billion in losses^9^. A study investigating the effect of this disaster on the cardiovascular system reported a more than threefold increase in the incidence of acute myocardial infarctions during the 6 years immediately post HK compared with pre-HK values^10^. Births in New Orleans, one of the most affected cities, declined by 30% in the decade spanning HK and has been proposed to be due in part to a reduction in female fertility rates alongside migration out of the region^11^. Adverse perinatal outcomes have been reported in women after exposure to HK. One prospective cohort study exploring exposure to HK reported an increased frequency of low birth weight (< 2500 g) and preterm delivery (< 37 weeks) in women who were early in pregnancy or conceived within 6 months after HK^12^. Other perinatal outcomes such as spontaneous abortions (SA) have also been reported to increase following natural disasters. An increased incidence of SAs were described after severe floods in Poland and New York; however, the studies included few numbers and were confined to small areas within the respective regions^13,14^.

In addition to perinatal outcomes, recent data has proposed that the acute stress caused by natural disasters could lead to in utero sex-specific effects, specifically alterations in secondary sex ratio (SSR), or the ratio of males to females at birth. A recent analysis comparing women exposed to a major earthquake in Chile during the first trimester of pregnancy versus controls found that stress exposure contributed to a decline in SSR, leading to birth of fewer male infants ^15^. A separate study of women who were exposed to the Great East Japan Earthquake of 2011 found reduced odds of delivering a male infant during stressful times, possibly due to increased spontaneous abortion of male fetuses^16^. While alterations of SSR occurring after natural disasters have been documented, the evidence favoring this phenomenon is weak and the cause still remains poorly understood.

Despite a large amount of data on the biological and psychological effects of natural disasters like HK, there has been little to no exploration on how these types of disasters alter reproductive physiology. Given the dramatic rise in utilization of assisted reproductive technology (ART) over the past decade and the increasing availability of national data from in-vitro fertilization (IVF) cycles, the effects of natural disasters on female reproductive function can be investigated further^17^. The objective of this study is to evaluate whether various IVF cycle parameters measured in individuals exposed to HK were more prone to negative alterations compared with those individuals not exposed to the deleterious effects of the hurricane. In addition, we aimed to investigate if pregnancies conceived via assisted reproductive technology (ART) in the post-HK period were more prone to adverse perinatal outcomes, miscarriage, or sex ratio disparities compared with those ART pregnancies established 18 months prior to HK. The national Society for Assisted Reproductive Technology Clinic Outcome Reporting System (SART CORS) database was used to identify women who underwent ART cycles during the time period immediately spanning HK in both affected and unaffected states.

## Material and methods

### Study population and design

After SART Research Committee approval, cycle data reported between January 2004 and December 2008 was retrospectively extracted from the SART CORS database, as this represented the timeframe surrounding the disaster. Of note, the dates used in the analysis are indicative of cycle start dates. Inclusion criteria were women 18 years and older undergoing fresh ART cycles that resulted in singleton pregnancies. We selected only singleton pregnancies since those with multiple gestations already have a heightened risk of pregnancy complications such as miscarriage, preterm delivery, and low birth weight infants. Patients who underwent third party reproduction, or cycles without recorded outcomes were excluded. For our study purposes, the timeframe from January 2004 to August 2005 accounted for the pre-HK period and the timeframe from September 2005 to December 2008 accounted for the post-HK period. The sixty-month period spanning Hurricane Katrina was chosen to account for women initiating an ART cycle prior to and after HK that resulted in a delivery. Affected women were categorized as those residing in the 5 FEMA designated states (Texas, Louisiana, Mississippi, Alabama, and Florida) and controls as those that resided outside FEMA zones.

### Outcome measures

The primary outcomes were incidence of full-term and preterm delivery, miscarriage rate, infant weight at birth, and infant sex ratio. Secondary outcomes were total follicle stimulating hormone (FSH) stimulation dosage and number of oocytes retrieved. Baseline characteristics such as age, maximum serum FSH level recorded, gravidity, and infertility diagnosis were also acquired.

Data on birth outcomes of IVF cycles were collected and verified by SART and reported to the Centers for Disease Control and Prevention in compliance with the Fertility Clinic Success Rate and Certification Act of 1992 (Public Law 102-493). The data in the SART CORS are validated annually with some clinics having on-site visits for chart review based on an algorithm for clinic selection. During each visit, data reported by the clinic were compared with information recorded in patients' charts. Ten of 11 data fields selected for validation were found to have discrepancy rates of ≤ 5% (10).

### Statistical analysis

A descriptive summary of baseline characteristics is presented as mean (SD) for continuous variables, median (interquartile range, IQR) for count variables and frequencies (%) for discrete variables. The comparisons of baseline characteristics as well as outcomes of interest were carried out using Generalized Estimating Equations (GEEs), with a suitable link function, in order to take into account the possibility of within patient correlation^18^.

In the comparison of outcomes of interest, prior specified baseline characteristics such as age (< 35, 35–37, 38–40, 41–42, > 42), maximum serum FSH level, gravidity, and infertility diagnosis (male factor, endometriosis, polycystic ovarian syndrome, diminished ovarian reserve, tubal factor, uterine factor, and unexplained) were included in the corresponding multivariable GEEs models to adjust for confounding factors. Cycle Start was used as the denominator in statistical analysis as our primary outcome measures were live births and miscarriage rates. We did not include cycle cancellations due to poor response in our study, as we focused on those women that had oocytes retrieved and embryos transferred, which are directly associated with our outcome measures. We chose the non-exposure dates specifically so that Cycle Start was up to the time of Hurricane Katrina, August 2005. Our exposure dates include all Cycle Starts from September 2005 to December 2008. That means the non-exposure period is 20 months and the exposure period is 40 months, total 60 months. The reason for the longer exposure period is that we accounted for the delays in care offered to IVF patients due to the clean-up process post hurricane. We doubled the exposure period from 20 to 40 months with the assumption that the first 12–18 months post hurricane would not be a reasonable time period to measure the possible impacts of stress and environmental aspects on couples starting an IVF cycle.

In order to assess the association between HK and outcomes of interest, an interaction term between time period, (before and after HK), and exposure [exposed (gulf states) and not exposed (all other states)] was included in the GEEs model along with the corresponding main effects and potential confounders. A significant interaction term in the model is regarded as an evidence of association between HK and outcome of interest. Effect estimates of time period (pre vs. post HK) by region (gulf or all other states) and interaction effect estimate along with 95% confidence interval (CI) are provided. A computed *p*-value ≤ 0.05 (two-sided) was considered statistically significant. Statistical analysis was performed using the SAS 9.4 software package (SAS Institute Inc., Cary, NC, USA).

## Results

Between January 2004 and December 2008, a total of 451,848 fresh autologous IVF cycles were recorded in SART CORS, leading to 190,624 pregnancies and 129,499 live births. Mean age of all women undergoing infertility treatment was 35.4 years old. Infant sex at birth and infant weight were available in 99.11% and 99.01% of cycles, respectively.

Univariate analysis of outcomes is presented in Table 1, grouped by exposed (gulf states) and unexposed (all other states) and then further subdivided by time period, pre and post HK. Patient age was found to be significantly different within unexposed states in the time period before and after HK, however, this difference was not clinically relevant, having an absolute difference of only 0.1 years of age. A similar effect was seen when maternal age was stratified as per SART. Maximum baseline FSH levels were higher in the time period after HK in both exposed and unexposed states, with an absolute difference of 0.4 and 0.6 ng/dL. The infertility diagnosis of diminished ovarian reserve (DOR) also increased in the time period after HK in both groups, as expected correlating with higher FSH levels.

**Table 1:** Univariate analysis of outcomes grouped by exposed (gulf states) and unexposed (all other states) and then further subdivided by time period, pre and post HK.

	n	Gulf states (exposed)			Other states (not exposed)		
		Pre-HK	Post-HK	P-value (Post-vs-Pre)	Pre-HK	Post-HK	P-value (Post-vs-Pre)
**Age**							
All, mean (SD)	451,848	34.7 (4.6)	34.6 (4.6)	0.37	35.4 (4.6)	35.6 (4.7)	< 0.0001*
< 35 (%)	186,780	7439 (48.6)	16,202 (47.5)	0.35	54,749 (42.0)	108,390 (40.1)	< 0.0001*
35–37 (%)	103,299	3500 (22.9)	8195 (24.0)		29,387 (22.5)	62,217 (23.0)	
38–40 (%)	91,989	2773 (18.1)	6249 (18.3)		26,937 (20.7)	56,030 (20.8)	
41–42 (%)	42,354	1065 (7.0)	2331 (6.8)		12,384 (9.5)	26,574 (9.8)	
> 42 (%)	25,409	536 (3.5)	1120 (3.3)		6921 (5.3)	16,832 (6.2)	
**Max FSH Level (mIU/mL), mean (SD)**		7.3 (3.7)	7.7 (3.7)	< 0.0001	7.7 (4.2)	8.3 (4.9)	< 0.0001*
**Gravidity, mean (SD)**		1.10 (1.41)	1.07 (1.40)	0.09	1.07 (1.37)	1.09 (1.39)	0.0003*
**Infertility diagnosis, (%)**							
Male	165,679	6877 (44.9)	14,735 (43.2)	0.003	46,690 (35.8)	97,377 (36.1)	0.22
Endometriosis	56,514	3038 (19.8)	5987 (17.6)	< 0.0001	17,068 (13.1)	30,421 (11.3)	< 0.0001*
PCOS	62,050	2458 (16.1)	5247 (15.4)	0.11	17,635 (13.5)	36,710 (13.6)	0.63
DOR	93,234	2882 (18.8)	6734 (19.8)	0.04	24,729 (19.0)	58,889 (21.8)	< 0.0001*
Tubal	81,273	3735 (24.4)	7156 (21.0)	< 0.0001	25,732 (19.7)	44,650 (16.5)	< 0.0001*
Uterine	22,868	823 (5.4)	1683 (4.9)	0.09	6605 (5.1)	13,757 (5.1)	0.76
Unexplained	55,376	846 (5.5)	2387 (7.0)	< 0.0001	17,010 (13.1)	35,334 (13.1)	0.79
**Total FSH Dosage, mean (SD)**		3046.5 (1442.8)	3145.2 (1483.8)	98.6 (66.2–131.0; < 0.0001)	3112.3 (1652.7)	3313.3 (1710.6)	^#^201.1 (187.4–214.8; p < 0.0001)*
**Number of Oocytes Retrieved, mean (SD)**		12.7 (7.7)	12.6 (7.8)	0.994 (0.981–1.008; 0.39)	12.5 (7.9)	12.4 (8.0)	^#^0.989 (0.984–0.994; p < 0.0001)*
**Clinical pregnancy, n (%)**							
Intrauterine	157,404	5682 (37.1)	13,217 (38.8)	1.07 (1.03–1.12; 0.001)	43,144 (33.1)	95,361 (35.3)	^#^1.10 (1.09–1.12; p < 0.0001)*
Biochemical	28,583	923 (6.0)	2286 (6.7)	1.12 (1.03–1.21; 0.01)	7907 (6.1)	17,467 (6.5)	^#^1.07 (1.04–1.10; p < 0.0001)*
**Pregnancy outcome, n (%)**							
Live Birth	128,889	4720 (30.8)	11,006 (32.3)	1.07 (1.03–1.12, 0.002)	35,363 (27.1)	77,800 (28.8)	^#^1.09 (1.07–1.10; p < 0.0001)*
Abortion	26,784	885 (5.8)	2024 (5.9)	1.03 (0.95–1.12; 0.50)	7249 (5.6)	16,626 (6.2)	^#^1.11 (1.08–1.15; p < 0.0001)*
Weight (g), mean (SD)		3189.1(628.6)	3174.8 (619.9)	-14.4 (-40.7, 12.0; 0.29)	3247.9 (615.9)	3234.8 (613.0)	^#^-13.1 (-22.5, -3.8; p = 0.01)*
Male sex (%)	43,817	1560 (51.0)	3624 (49.9)	0.96 (0.88–1.04; 0.33)	11,795 (49.6)	26,838 (50.3)	^#^1.03 (1.00–1.06; p = 0.06)

*Significant p-value.

^#^Odds ratios with 95% CIs were given for the outcome variables (total FSH dosage, number of oocytes retrieved, clinical pregnancy, pregnancy outcome, weight, and male sex) in addition to the p-values.

Multivariable analysis adjusting for age, maximum FSH levels, gravidity, and infertility diagnosis is presented in Table 2. An additional column labeled “Interaction Effect Estimate” is included to quantify the association between HK and the various outcomes of interest. After controlling for a priori specified potential confounders, our results demonstrate no association between HK and clinical pregnancy rate, live birth rate, or overall preterm delivery rate (< 37 weeks) in women with singleton pregnancies conceived after ART. In addition, when preterm delivery was further stratified based on gestational age (< 37, < 32, 28–32, and < 28 weeks), changes in the rates of preterm delivery were not associated with HK in any given subgroup. Differences in other perinatal outcomes such as rate of SA or infant weight at birth in the exposed and unexposed groups were also not associated with HK.

**Table 2:** Multivariable analysis adjusting for age, maximum FSH levels, gravidity, and infertility diagnosis.

	Gulf states (exposed)			Other (not exposed)			Interaction effect estimate (CI, p-value)
	Effect Estimate/OR^#^ (Post-vs-Pre)	95% CI	P-value	Effect Estimate/OR^#^ (Post-vs-Pre)	95% CI	P-value	
**Total FSH Dosage, effect estimate**	39.5	3.7–75.3	0.03	171.2	156.3–186.1	< 0.0001	− 116.9 (− 115.8, − 78.0; p < 0.0001)*
**Number of Oocytes Retrieved, effect estimate**	1.017	1.001–1.032	0.03	1.010	1.005–1.020	0.0001	1.007 (0.991–1.023; p = 0.40)
**Gestational Age (Weeks), ORs**							
Overall (> 20 weeks)	0.63	0.08–5.23	0.67	0.96	0.53–1.71	0.88	0.62 (0.06–6.02; p = 0.68)
< 37 weeks	1.02	0.88–1.18	0.82	1.03	0.98–1.08	0.29	0.99 (0.85–1.15; p = 0.87)
< 32 weeks	1.26	0.92–1.73	0.15	1.00	0.89–1.13	0.96	1.26 (0.90–1.77; p = 0.18)
28–32 weeks	1.17	0.77–1.77	0.46	0.90	0.77–1.05	0.17	1.32 (0.85–2.06; p = 0.21)
< 28 weeks	1.38	0.85–2.23	0.19	1.17	0.97–1.42	0.10	1.16 (0.69–1.95; p = 0.58)
< 20 weeks	0.99	0.89–1.10	0.82	1.02	0.98–1.06	0.26	0.97 (0.87–1.09; p = 0.65)
< 12 weeks	0.91	0.70–1.20	0.51	1.05	0.96–1.14	0.30	0.86 (0.65–1.14; p = 0.31)
**Clinical pregnancy, OR**							
Intrauterine	1.12	1.06–1.18	< 0.0001	1.17	1.15–1.19	< 0.0001	0.95 (0.91–1.01; p = 0.09)
Biochemical	1.07	0.97–1.18	0.19	1.08	1.05–1.11	< 0.0001	0.98 (0.89–1.09; p = 0.75)
**Pregnancy outcome, OR**							
Live Birth	1.11	1.06–1.17	< 0.0001	1.15	1.13–1.17	< 0.0001	0.96 (0.91–1.02; p = 0.19)
Abortion	1.06	0.96–1.17	0.24	1.14	1.10–1.17	< 0.0001	0.94 (0.85–1.04; p = 0.24)
Infant Weight, effect estimate	− 39.8	(− 72.0, − 7.6)	0.02	− 14.9	(− 25.7, − 4.2)	0.01	− 25.1 (− 59.0, 8.8; p = 0.15)
Male Infant Sex, OR	0.92	0.83–1.03	0.14	1.03	1.00–1.07	0.09	0.90 (0.81–1.01; p = 0.07)

*Significant p-value.

^#^*OR* odds Ratio.

IVF cycle parameters such as total FSH dosage required and number of oocytes retrieved were analyzed secondarily (Table 1). Although higher doses of gonadotropins were used in the post HK period in both groups, unaffected states increased utilization of those gonadotropins more so than affected states, and this was in part associated with HK itself (p < 0.0001). In other words, even though affected states increased utilization of gonadotropins over time, their utilization of these medications did not increase as much as the unaffected states did. Number of oocytes retrieved was not found to be altered by the effect of HK after multivariate analysis.

Details regarding the association between HK and SSR are demonstrated in Table 2. The proportion of male infants born was similar pre and post HK in unaffected regions (49.6% vs. 50.3%). However, in FEMA designated states, comparison of pre and post HK SSRs revealed fewer males were born after HK (51.0% vs. 49.4%). In order to evaluate if HK contributed to this shift, an interaction effect estimate was calculated and found to be 0.90 (CI 0.81–1.01; p = 0.07). Although not statistically significant, there is a clear trend showing that the decreased births of male infants were at least in part associated with exposure to HK. Differences in other perinatal outcomes between exposed and unexposed states in pre and post HK periods were also analyzed. Birth weight, preterm delivery, live birth rate, and miscarriage rate did not appear to be associated with HK (p = 0.15, 0.87, 0.19, and 0.24, respectively). Clinical pregnancy rate increased in areas unaffected by HK exposure in the pre vs. post periods, 33.1 vs. 35.3 (p < 0.0001). This increase was also observed to occur in FEMA designated areas, 37.1 vs. 38.8% (p = 0.001); however, the difference was not as substantial, interaction effect estimate 0.95 (CI 0.91–1.01; p = 0.09).

## Discussion

This is the largest study to date evaluating the association between natural disasters and pregnancy outcomes and the first study to specifically examine the effects of HK on ART pregnancy outcomes and sex ratio disparity. Prenatal maternal exposure to extreme stress caused by natural disasters has previously been associated with adverse pregnancy outcomes such as reduced infant weight and increased risks of preterm delivery^19^. In addition, SA has been reported to occur more frequently in pregnant women who are exposed to stressful environments.

Analysis of ART treatment outcomes revealed that the clinical pregnancy rate increased in patient populations post HK as expected with improving IVF techniques over time. However, the increase was greater in those patients residing in areas not exposed to HK compared to those exposed, 2.2% vs. 1.7% respectively. FEMA designated areas failed to achieve the same escalation in ART success that the rest of the country observed. An interaction effect estimate model was again used to determine if the effect observed was in part due to exposure to HK. There was a trend observed, however the effect did not achieve significance (CI 0.91–1.01; p = 0.09). This association was not seen when analysis was focused on live birth outcomes, interaction effect estimates 0.96 (CI 0.91–1.02; p = 0.19).

Alterations in SSR at birth has been a fascinating topic in the literature over the past few decades. Arguments suggest that in certain situations, male fetuses are more likely to abort then females. Although the biological mechanisms are unclear, it is been postulated that male fetuses are more sensitive to stress than their female counterparts. This increase in sensitivity potentially leads to a higher proportion of male fetal abortuses compared with females. In fact, studies have shown that women who experience severe stress while pregnant, such as from natural disasters, terrorist attacks or mass layoffs, have lower than expected male to female SSRs ^20,21^. One possible mechanism that explains the cause of altered SSR is that less robust male fetuses may be preferentially aborted, allowing for natural selection of stronger male infants who are more apt to survive under stressful conditions^15^. Alternatively, it is plausible that high stress environments lead to an asymmetrical selection of female fetuses which would be required for perpetuation of the species post-disaster. Our data confirms that there may be a trend toward declining male to female SSR in populations exposed to high stress such as those experienced with HK. While our data confirms that there may be a trend toward declining male to female SSR in populations exposed to high stress, the sex ratio was not significantly altered. Exposed populations had a reduced rate of male births post HK compared with an unchanged rate of male birth in populations not exposed to HK, interaction effect estimate 0.90 (CI 0.81–1.01; p = 0.07). The trend indicates delivery of male infants occurs less frequently and adds weight to the theory behind alterations in SSR during maternal exposure to extreme stressful environments.

Pregnancies conceived using ART provide a unique study population because the gestational age is accurately documented, and this is paramount when analyzing pregnancy outcomes such as preterm delivery and SAs. We used cycle data reported to the national SART CORS database to collect delivery information on singleton pregnancies in order to avoid the effect that multiple gestations may have on gestational age at time of delivery, as these pregnancies are already at high risk of early delivery. One of the major strengths of this analysis is the large sample size. We include a database with a sample of over 400,000 cycles with accurately reported gestational ages at time of delivery, compared with other studies that use less precise means of documenting pregnancy outcomes.

The SART database includes ART data from 85% of the centers within the US and is prospectively collected by each individual center, thus not all ART data is available for analysis. Despite the data being validated by SART, certain outcomes such as birth weight are subject to recall bias since they are primarily self-reported to clinics by patients. Other possibly useful parameters such as body mass index and ethnicity were not universally available for the time periods analyzed, not reported in 74.2% and 35.1% of the data respectively. Markers of stress levels were also not collected in the SART CORS database, and thus could not be formally evaluated. There are other limitations within this study that complicate the interpretation of the results, such as lack of adjusting for multiple comparisons. Loss of general medical follow up could be greater after a disaster leading to fewer available SART reported cycles post-HK compared with pre-HK. The effects of the environment and local infrastructure on ART, and changes in patient population driven by economic hardship, could not be evaluated using this dataset. HK did not affect the 5 states uniformly; however patient specific information based on zip codes are not available for research using SART CORS. As with many natural disasters, there is often a large displacement of individuals by population out-migration and infrastructure impact^11^. Thus, some individuals who were invariably affected by HK may have sought ART treatment at clinics outside of FEMA zones. Even though those individuals were exposed to HK, their cycle parameters and outcomes would have been reported as if they resided in unexposed states. Patients who failed to conceive initially could also have potentially been represented more than once if they initiated a cycle before and after HK. Pregnancy data was limited to singleton gestations to minimize confounders; however, this could result in limited information on pregnancies as a whole. In addition, we could not account for other disasters occurring during the same time period. For example, Hurricane Rita which was a Category 5 hurricane, made landfall in the gulf states on September 2005. Lastly, embryo quality parameters and mode of insemination were not provided as part of this dataset since this type of data was not gathered by SART at the time of HK.

In summary, we failed to identify a link between HK and the rate of the perinatal outcomes analyzed, the rate of preterm delivery, or the rate of spontaneous abortion. In regard to previous reports that state extreme stress leads to a reduced male to female secondary sex ratio, we can only report that our data indicate a trend towards less male infants born, but this association was not statistically significant.
